# Research on Community Detection in Complex Networks Based on Internode Attraction

**DOI:** 10.3390/e22121383

**Published:** 2020-12-07

**Authors:** Jinfang Sheng, Cheng Liu, Long Chen, Bin Wang, Junkai Zhang

**Affiliations:** School of Computer Science and Engineering, Central South University, Changsha 410083, China; jfsheng@csu.edu.cn (J.S.); awesome@csu.edu.cn (C.L.); lgchen@csu.edu.cn (L.C.); zhangjunkai@csu.edu.cn (J.Z.)

**Keywords:** community detection, complex networks, node attraction, local information, important nodes

## Abstract

With the rapid development of computer technology, the research on complex networks has attracted more and more attention. At present, the research directions of cloud computing, big data, internet of vehicles, and distributed systems with very high attention are all based on complex networks. Community structure detection is a very important and meaningful research hotspot in complex networks. It is a difficult task to quickly and accurately divide the community structure and run it on large-scale networks. In this paper, we put forward a new community detection approach based on internode attraction, named IACD. This algorithm starts from the perspective of the important nodes of the complex network and refers to the gravitational relationship between two objects in physics to represent the forces between nodes in the network dataset, and then perform community detection. Through experiments on a large number of real-world datasets and synthetic networks, it is shown that the IACD algorithm can quickly and accurately divide the community structure, and it is superior to some classic algorithms and recently proposed algorithms.

## 1. Introduction

Community structures exist in many networks in the real world, such as social networks, e-commerce networks, biological networks, routing networks, and so on. In recent years, community structure detection of complex networks has attracted much attention, and a series of complex network mining algorithms have been proposed [1,2,3,4,5]. The identification of community structure in the network has great application value. For example, discovering organizations and providing personalized services in social networks; precision marketing and intelligent recommendation in e-commerce networks; it can also be used to discover the close relationship between proteins in biological networks and so on. The community detection methods have been widely used in various disciplines and many fields. So far, researchers have proposed a large number of community structure detection methods from different perspectives, such as graph partitioning [6], modularity optimization [7], and dynamic methods [8,9]. However, most existing algorithms need to consider setting parameters, so the quality of identifying communities depends largely on the choice of parameters. For example, Markov clustering algorithm (MCL) [10] is a dynamic method that is widely used in graph clustering studies, but MCL is more sensitive to setting parameters. In addition, many algorithms also have problems with unstable detection results. For example, the label propagation algorithm (LPA) [11] and fluid community method (FluidC) [12] although their complexity are relatively low and the calculation speed are fast, they may produce different division results. In addition, the increasing network scale also poses another challenge to community detection. Compared with some algorithms that have better detection results on small networks, such as the Girvan and Newman algorithm (GN) [13]. Due to the high time complexity, it cannot be used in large-scale networks. Therefore, for many existing complex network community detection algorithms, they cannot fully meet the needs of various scenarios in the real world.

Aiming at the above problems, this paper proposes a new community detection algorithm named IACD. Firstly, the degree and the k-core value [14] are used to re-define the importance of nodes. Then, based on the idea of the interaction between celestial bodies in physics, the relationship of gravitational interactions between pairs of nodes in the network is constructed, so that each node chooses another node that is most attractive to it, forming an attractive node pair. Finally, all the nodes will find the matching nodes, and the divided community structure will be obtained.

The main contributions of this work are as follows: (1) A new method based on physical phenomena: the IACD draws on the idea of gravitation in physics and defines the attractiveness calculation method between nodes in the network. It can intuitively discover communities and be understood easily; (2) Parameter free: IACD does not rely on parameter setting and can automatically perform communities division based on the local topology of the network; (3) Scalability: IACD uses second-order neighbors of nodes to construct attractive node pairs which reduces the calculation cost. Therefore, the IACD algorithm has low time complexity and can be applied to large-scale networks; (4) Higher accuracy: A large number of experiments on real-world and synthetic networks show that the IACD has a good ability to divide communities.

## 2. Related Work

At present, there are many algorithms in the field of complex network community detection. This section mainly introduces and analyzes several types of representative algorithms. The method of dividing the community structure according to the algorithm is roughly divided into graph partitioning, hierarchical clustering, label propagation, and modularity optimization, and other community detection algorithms.

Graph partitioning methods. Graph cut [13] is a classic problem. The idea is to divide the network graph into two subgraphs first, then separate the two subgraphs, and iterate repeatedly until the required number of subgraphs is obtained. For the graph partitioning methods, there are two points to note. One is to know the number of communities in advance, and the other is to know the number of nodes that should be included in each community. The representative algorithm of graph partitioning algorithm is Kernighan-Lin (KL) [15] algorithm, which is a tentative dichotomy algorithm based on the idea of greedy optimization. The KL algorithm needs to introduce a gain function Q in the process of dividing communities. The value of Q is defined as the difference between the number of all edges in two communities and the number of connected edges between the two communities.

Hierarchical clustering methods. The idea of hierarchical clustering algorithm is to cluster nodes by layer-by-layer iteration to achieve community detection of the network. According to the method of hierarchical decomposition, it can be divided into splitting method and agglomeration method. The splitting algorithm is a community segmentation from top to bottom. The algorithm first considers the entire network as a community, and then divides the community into multiple small communities through layer-by-layer recursive segmentation. A typical splitting algorithm is the GN algorithm [13]. This algorithm proposes the concept of edge betweenness, which refers to the number of all shortest paths in the network through the current edge. Because the shortest paths of nodes in different communities in the network can only be obtained by the edges between communities, the connected edges between communities often have a large edge betweenness. The other is the agglomeration algorithm, which initially treats each node in the network as a community alone, and then performs layer-by-layer aggregation iterations. For example, Newman proposed a fast and complex network community detection algorithm based on local search, referred to as FN algorithm [16].

Label propagation methods. The label propagation algorithm is a graph-based semi-supervised learning algorithm. The main idea is to use the label information of the labeled nodes to predict the label information of the nodes that have not been labeled. Due to the advantages of label propagation algorithms in terms of time complexity, more and more researchers have invested in that, and have successively proposed label propagation algorithms with various ideas. The most classic label propagation algorithm among these algorithms is LPA proposed by Raghavan [11] et al. The LPA algorithm initially assigns unique labels to each node, and then for each node, it selects the label of the node with the highest frequency of labels in the neighboring nodes to update to its own label. Iteratively update the node label until the maximum number of iterations is reached or the label of each node in the network no longer changes, and finally the nodes with the same label will be assigned to the same community.

Modularity optimization methods. The main idea of the modularity optimization algorithm is to optimize the modularity index Q by defining the objective function of the quality of community division. Therefore, the larger the community modularity value after the division, the better the division result is. However, because there are many ways to divide the graph, it is not feasible to use the exhaustive method to achieve the optimal modularity, and the optimization of modularity is an NP-complete problem [17]. Researchers have devised many methods to efficiently obtain their approximate solutions, such as Fastgreedy [18], Fiduccia-Mattheyses [19], Louvain [20] and other methods. But, the research results show that this type of methods have resolution limitation problems on a large number of real networks [21], especially on large-scale networks.

The IACD algorithm based on internode attraction proposed in this paper has a good performance in real-world network datasets and synthesis networks. It can run on large-scale networks effectively.

## 3. The IACD Method

### 3.1. Preliminaries

Community structure detection is the process of partitioning communities in the network. We model a dynamic network as an undirected graph G=(V,E), where *V* is the set of nodes and *E* is the set of edges. The neighbors of a node *v*∈*V* in an undirected graph *G* is the set N(v)={x∈V|(v,x)∈E} containing all nodes directly connected to it. Furthermore, A node in N(v) has a set of nodes with connected edges, excluding node *v* itself. The set of second-order neighbor nodes of node *v* is denoted as N2(v)={x∈V|(n,x)∈E,n∈N(v)}. Let SL(v,m) represents the shortest path length between nodes *v* and *m* in the network. Let KScore(v) represents the global position of *v* node in the network, which is defined using the k−core (also called k−shell) decomposition analysis [14]. The larger the value, the closer the node is to the center of the network. Before explaining our proposed algorithm in detail, we formalize some basic definitions. All important definitions are summarized and briefly defined in Table 1.

**Definition** **1.** (Node Influence) The node influence IF(v) reflects the influence of node v in the entire network, considering the location and degree of the network in which it is located.

(1) IF(v)=Deg(v)×KScore(v)

Here, in order to take into account both the local influence of the node and the position of the node in the entire network, we consider both the degree value Deg(v) and the position of the node in the network, i.e., the k-core value KScore(v).

**Definition** **2.** (Global Influence of Nodes) Normalize the influence IF(v) of node v.

(2) $$GIF\left(v\right)=\frac{IF(v)}{MAX\_IF}$$

Here, MAX_IF is the influence value of the node with the largest node influence in the network.

**Definition** **3.** (Triangle) Triangles have stability. Triangle(v,m) represents the number of triangles formed by node v, node m, and other nodes. The larger the number, the closer the two are.

(3) Triangle(v,m)=|N(v)∩N(m)|

**Definition** **4.** (Coefficient of Attraction) P(v,m) represents the attractive coefficient of node m to node v in the node pair formed by node v and node m.

(4) $$P\left(v,m\right)=\frac{Triangle(v,m)+1}{Deg(v)}$$

Here, P(v,m) may not be equal to P(m,v), the formula is directional, because Deg(v) may not be equal to Deg(m).

**Definition** **5.** (Internode Attraction) In the node pair formed by node v and node m in the network, the attraction Attr(v,m) of node m to node v is defined as follow.

(5) $$Attr\left(v,m\right)=P\left(v,m\right)\frac{GIF(v)\times GIF(m)}{SL{\left(v,m\right)}^{2}}$$

Here, we only consider nodes in the second-order neighborhood of node *v*, i.e., N2(v), and for nodes outside the second-order, the internode attraction will be very small due to the distance. Besides, the formula is directional, Attr(v,m) may not be equal to Attr(m,v), because P(v,m) may not be equal to P(m,v).

### 3.2. The IACD Model

**Figure 1:**
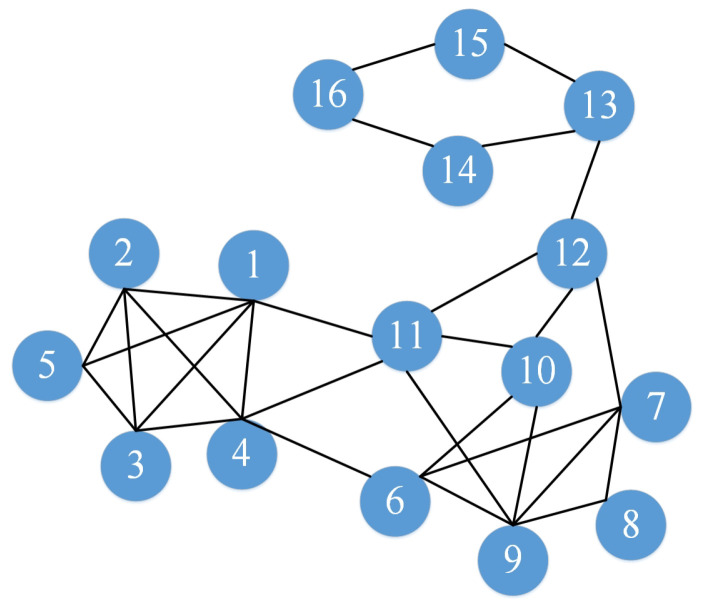
An example network.

At present, many scholars have proposed various complex network community detection algorithms from different perspectives, each with advantages and disadvantages. The community detection algorithm IACD based on internode attraction, the main idea of this algorithm consists of three steps: node importance representation, attractive node pair selection and community division. The IACD model is illustrated in detail, using the example network shown in Figure 1.

Node importance representation.In the field of important node selection of complex networks, there are many methods to describe the importance of nodes, such as degree centrality [22], closeness centrality [23], betweenness centrality [24], k-core [14], and subgraph centrality [25], leader rank [26], page rank [27], hits [28], etc.

However, these indicators cannot fully reflect the importance of a node in the network. For example, a node with a high degree value is often considered an important node. But, if the position of this node is at the edge of the network, it may not be as important as a node with a low degree value but located at the core position. We comprehensively consider the two factors of the node’s own degree value and its location, and then redefine the importance expression method of node *i* and record it as the node influence IF(i) according to Equations (1). After normalization, we obtain the global influence GIF(i) according to Equations (2). For the example network in Figure 1, the influence of each node is shown in Table 2.

Attractive node pair selection. We believe that there is attraction between the nodes in the network. Drawing on the gravitational relationship between cosmic celestial bodies in physics, we define the gravitational interaction Attr(i,j) between nodes *i* and *j* in the network. That is, the attraction of node *j* to node *i* is directly proportional to the product of the global influence of the two nodes, inversely proportional to the square of the two paths length, and proportional to the attraction coefficient of node node *j* to node *i*. For node *i*, we consider selecting its most attractive node from its second-order neighbor N2(i) to form an attractive node pair. Consider the strength of the attractive effect first, if there are nodes with equal attractive strength, then consider the node *j* with the shortest path length. Of course, there may be multiple nodes that meet the conditions. At this time, one can be randomly selected, that is, for node *i*, there is only one node *j* that has the strongest attraction to it, we call node *j* as MAX_Strong(i). MAX_Strong(i) defined as Equation (6).

(6) MAX_Strong(i)=maxAttr(i,j)andminSL(i,j),j∈N2(i)

For node $v_{1}$ in Figure 1, the second-order neighbor N2(i) of node $v_{1}$ is {2,3,4,5,6,9,10,11,12}. For these 9 points, the attractiveness to node $v_{1}$ is calculated in the Table 3.

It can be judged that the most attractive node for node $v_{1}$ is node $v_{4}$, so node $v_{1}$ and node $v_{4}$ form an attractive node pair $\left[v_{1},v_{4}\right]$ according to Equation (6). For each node in the network, there will be a node with the strongest attraction to form an attractive node pair.

Community division.In the second stage, each node will find a node that is most attractive to it, forming an attractive node pair. In the third stage of community division, we first obtain that each node forms an attractive node pair and then forms a two-dimensional table structure. For the example network in Figure 1, the two-dimensional table is shown in Table 4.

**Figure 2:**
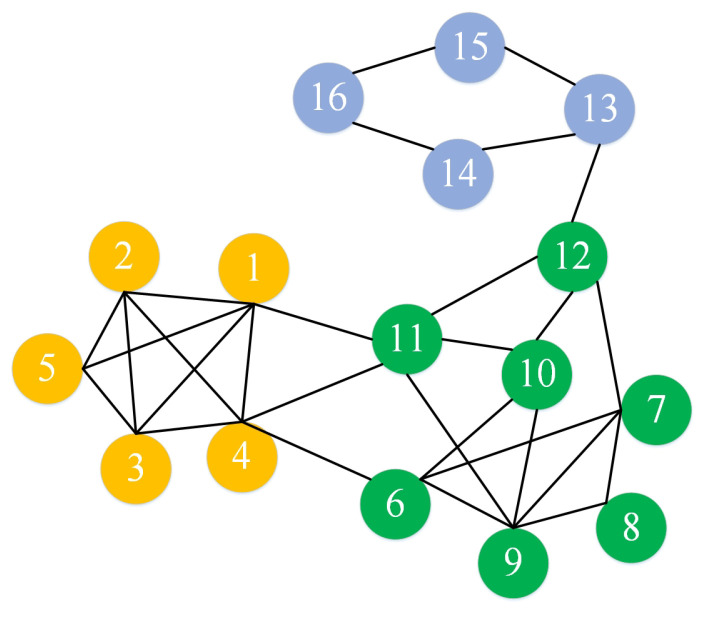
Example network community detection result.

Then we do the community division. First, each node can be regarded as a community individually. We start with the head node *i* in two-dimensional table to determine whether the corresponding most attractive node *j* is in the same community. If the two points are in the same community, then the next set of node pairs will be judged. Else, all nodes of the community to which *j* belongs are merged with the community to which *i* belongs to form a new larger community. Repeat this process until all nodes execute this process. In Table 4, we can observe that the strongest attraction for node $v_{1}$ is node $v_{4}$, so divide node $v_{4}$ and node $v_{1}$ into the same community C1{1,4}. The strongest attraction for node $v_{2}$ is node $v_{1}$. Since the community to which node $v_{1}$ belongs does not include node $v_{2}$, then merge the two communities and update C1 to {1,4,2}. For node $v_{3}$, node $v_{1}$ is not in the same community, then update C1 to {1,4,2,3}. Then for node $v_{4}$, since node $v_{1}$ has been merged with node $v_{4}$ into the same community before, skip this group, continue to judge the next group of nodes, and repeat this process until all ends. Finally, we can get Three communities C1{1,2,3,4,5}, C2{6,7,8,9,10,11,12} and C3{13,14,15,16}. The result is shown in Figure 2.

### 3.3. The IACD Algorithm

First, according to Equations (1) and (2), the IF and GIF of each node are calculated in turn. Then calculate the strongest attractive node of each node according to Equations (5) and (6) to form attractive node pairs. Finally, divide the two-dimensional table and output the result. The IACD algorithm is given in Algorithm 1.
**Algorithm 1** Community Detection Based on Internode Attraction.**Require:**   Graph:G=(V,E);**Ensure:**
   C, the graph G’ communities   // Initialization of the information of each node.   **for** each node i in V **do**
      compute the IF value using Equation (1)   **end for**   **for** each node i in V **do**
      compute the GIF value using Equation (2)   **end for**   // Node attraction judgment.   **for** each node i in V **do**
      **for** each node j in N2(i) **do**
         compute the Attr(i, j) value using Equation (5)      **end for**      choose the Max_Strong(i) value using Equation (6)   **end for**   // Communities division process.   Construct attraction two-dimensional table.   **for** each pair of nodes in table **do**
      merge the two communities of each node belongs to   **end for**

### 3.4. Time Complexity

The whole algorithm is mainly divided into three phases. In the first stage, determine the influence and global influence of each node, the time complexity is O(n)+O(e), *n* is the number of nodes in the network, and *e* is the number of edges; in the second stage, each node calculates the attraction value with its second-order neighbor nodes, and form the node pair with the most attraction. The time complexity is $O(n<k{>}^{2})$, and <k> is the average degree. Finally, the community division is performed, the time complexity is O(n).

In summary, the time complexity of the IACD algorithm is the sum of the three parts: $O\left(n\right)+O\left(e\right)+O\left(n<k{>}^{2}\right)+O\left(n\right)\approx O\left(n<k{>}^{2}+e\right)$.

## 4. Experimental Evaluation

In this section, we compare our proposed algorithm IACD with several representative algorithms on synthetic and real-world networks to study the performance on community detection.

### 4.1. Data Description

Synthetic networks. To generate artificial synthetic networks that are more similar to real-world networks, we use the well-known benchmark generation model LFR [29] to generate network datasets. The LFR model was proposed by Fortunato et al. It can easily control the LFR model to create a synthetic network by using several properties of the network in real life as parameters. For example, the number of nodes in the network, the average degree, the maximum degree, the size of the community, and the average clustering coefficient. The most important parameter is the mixing parameter mu, which is defined as the ratio of the number of consecutive edges of a node outside the community to the total number of edges. The larger mu indicates that the more connected edges outside the community, the more difficult the identification of the community. The main parameters are shown in Table 5. In order to evaluate the quality of the IACD algorithm more effectively. First, we fix the number of nodes N=1000, the average degree <k> =15, the maximum degree maxK=50, the minimum community size minC=20, and the maximum community size maxC=50. Then change the parameter mu form 0.1 to 0.7 to generate several networks. In addition, in order to evaluate the community recognition effect of community detection algorithms with different network densities. We fixed the mixing parameter mu=0.2 and then changed the average degree <k> from 10 to 35 and maxK=5×K to generate multiple synthetic networks with different densities. Finally, in order to evaluate the effect of community detection algorithm with different node sizes. We fixed mu=0.1, average degree <k> =15, maximum degree maxK=50, and change *N* from 1000 to 30,000 with step size around 5000 to generate multiple synthetic networks with different scales. Besides, we also generate a very large-scale network, which contains 500,000 nodes. The purpose is to explore the performance of IACD on very large-scale synthetic network.

Real-world datasets. The experiment selects some real-world networks with real community structure. The basic statistics of the dataset are shown in Table 6.

Zachary’s karate network [30]: This is a well-known American karate club network, which represents the friendship between 34 members of the club. For some time, due to the issue of leadership disagreement, the network was divided into two communities.

Amazon network [31]: This network is a record of purchases on the amazon website for a period of time. It contains 925872 edges and 334863 nodes. In this network, products represent nodes, and the products that are frequently co-purchased are represented by edges, and the category to which each product belongs is considered as a real community.

Politics books network [32]: This is an American political books network, which consists of 105 nodes and 441 edges. Among them, nodes represent books, and the relationship between books that are frequently purchased by the same buyer is represented by edges.

Dolphins network [33]: This network is a social relationship network between dolphins. Frequent exchanges between dolphins constitute a connection between different dolphins, consisting of 62 nodes and 159 edges. Nodes represent dolphins, and edges represent interactions between dolphins.

AGBlogs network [34]: This network is a network of political blogs. It contains 1222 points and 33,428 edges, including two real community structures.

UmbcBlog network [35]: This network contains 2382 edges and 404 nodes, including two real community structures.

### 4.2. Comparing Algorithms

To evaluate the performance of IACD algorithm, we compare it with several representatives algorithms which belong to different viewpoints and different periods. The basic idea and time complexity of them are shown in Table 7.

LPA [11]: LPA is a very fast community detection method implemented by label propagation. Due to its linear time complexity, it can run on large-scale networks.

Fastgreedy (FG) [18]: FG is a modularity optimization algorithm, which uses the greedy method to optimize the modularity to reveal the community structure of the network.

Louvain [20]: Louvain is a well-known multi-level modularity optimization algorithm. Compared with the modularity algorithm proposed by Newman [36], it has lower time complexity.

GN [13]: The GN algorithm continuously deletes the edges with the largest betweenness relative to all source nodes in the network, and then recalculates the betweenness of the remaining edges in the network relative to all source nodes, repeating this process until all edges are deleted.

FluidC [12]: FluidC is a scalable propagation-based community detection algorithm that simulates the expansion and contraction behavior of fluid until a balance is found.

Leading-Eigenvector (LE) [37]: LE finds the community structure in the network by constructing the Laplacian matrix feature vector of the network.

CDID [38]: CDID was proposed in 2019. It considers the network as an adaptive dynamic system, then simulates the information interaction on it, and reveals the community structure in the network through information dynamics.

### 4.3. Evaluate Metric

In the research of non-overlapping community detection in complex networks, many standards have been proposed to quantify the quality of community detection. For example, Modularity [39], Normalized Mutual Information(*NMI*) [40], Adjusted Rand Index(*ARI*) [41], *Purity* [42]. Here, we briefly introduce three evaluation metrics used in this paper.

*NMI* is a similarity measurement method which assumes that if two partitions are more similar, less additional information is needed to infer the allocation of the other partition. It is defined as follow:

(7) $$NMI\left(X;Y\right)=\frac{2I(X;Y)}{H(X)+H(Y)}$$

Here, the range of *NMI* value is 0 to 1. The larger the *NMI* value, the more similar the two parts are.

*ARI* is another viewpoint measure of similarity to compare two clusters, defined as follow:

(8) $$ARI=\frac{RI-ExpectedRI}{MaxRI-ExpectedRI}$$

Here, RI is a method of similarity measure, which considers all sample pairs. The quality of community detection is evaluated by calculating the number of identical and different sample pairs in the predicted community partition and the true community partition. The larger the *ARI* value, the more similar the two parts are.

*Purity* is the percentage of correctly classified samples to the total number of samples. The larger the *Purity* value, the more similar the two parts are. It is defined as follow:

(9) $$Purity\left(\Omega ,C\right)=\frac{1}{N}\sum_{i=1}^{k}max_{j}\left|\omega_{i}\cap c_{j}\right|$$

Here, $\Omega =\{\omega_{1},\omega_{2},\ldots ,\omega_{k}\}$ is the set of predicted community divisions, $C=\{c_{1},c_{2},\ldots ,c_{j}\}$ is the set of true classifications, and *N* is the total number of samples.

### 4.4. Performance Evaluation

This section mainly evaluates the partitioning ability of the community detection algorithm IACD. The comparison algorithm and experimental data used in the experiment are as described in the previous section. The evaluation metrics are *NMI*, *ARI*, and *Purity*. Most algorithms are implemented with python packages such as sklearn-cluster, igraph, and other comparison algorithms use the source code provided by the original paper. The comparison algorithms for small and medium scale networks are run 50 times on each experimental dataset, and the results are averaged 30 times on large networks. All experiments are run on a desktop computer. We rendered experiments on Feiteng Server provided by Kirin Operating System, Feiteng 1500 A CPU, 32 G memory and 2TB hard disk.

#### 4.4.1. Performance Evaluation on Real-World Datasets

In seven real-world networks with node size ranging from 34 to 334,386, we use 7 different comparison algorithms and three evaluation metrics for comparison. The experimental results are shown in Table 8. As a whole, in real networks, the comparison between the results of the algorithms on the *purity* evaluation metrics is not significant enough, and the two metrics *NMI* and *ARI* are more representative of the results.

**Figure 3:**
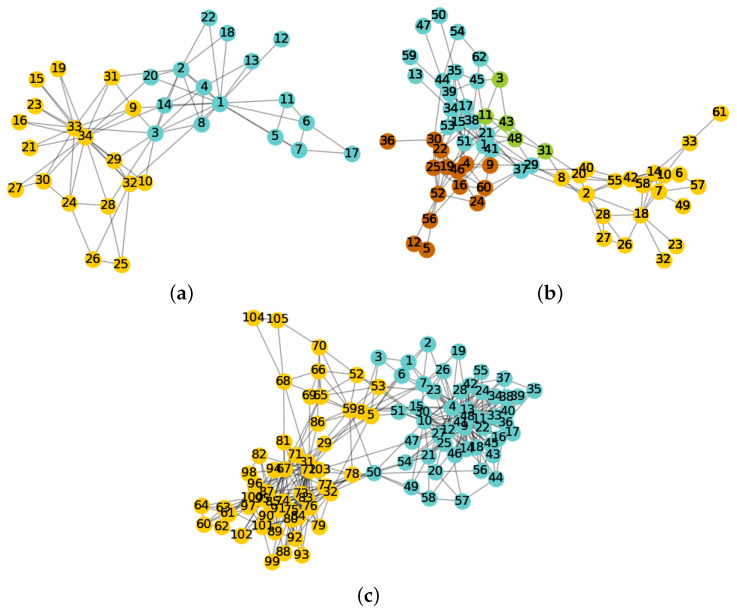
The community detection result. (**a**) Community detection result on Karate. (**b**) Community detection result on Dolphins. (**c**) Community detection result on PolBook.

Karate network. The IACD algorithm proposed in this paper is far more than the other seven algorithms in both the *NMI* metric and the *ARI* metric, and the three metrics have achieved maximum values. The visualization of community detection results on the Karate network is shown in Figure 3a. We can observe that the IACD algorithm completely separates the network and is consistent with the real structure. Except for the CDID algorithm, the results of the other six algorithms are lower than 0.80 on each metric.

Dolphins network. The LPA algorithm achieves the highest value on both *NMI* and *ARI*. The IACD algorithm reach 0.63 on *NMI*, which is only 0.02 from the first place, and ranked third in all algorithms on the *ARI* metric. Our algorithm performs well in general and is superior to most comparison algorithms. The visualization of the partition results is shown in Figure 3b. Our algorithm divided the network into four communities. One of the communities contains only five nodes, indicating that the IACD algorithm can identify small structures in the network and is more authentic. It can be seen that the algorithms such as LE and CDID have lower values and perform worse in the dolphin network from Table 8.

Polbook network. IACD algorithm achieves the highest value in the *NMI* index, reaching 0.61, and the difference between the *ARI* index and the highest value obtained by the GN algorithm is only 0.01. The visualization of the division results is shown in Figure 3c.

AGBlogs network and UmbcBlog network. The IACD algorithm achieves the highest values in both *NMI* and *ARI* metrics, and the quality of the division results is very high. It can be observed that the detection results of algorithms such as LE and Louvain are also very good, and there is not much difference with IACD, but the value of the metrics of community detection by algorithms such as GN and CDID is low.

In order to evaluate the community detection performance of the IACD algorithm on large-scale real-world network, we used amazon network for experimental evaluation. Due to the high complexity of the GN algorithm, it cannot run on this network. LPA algorithm achieves the highest value on *NMI*, IACD is 0.51, the difference is only 0.01. The LE algorithm partition result is not ideal, and the *NMI* value is only 0.01. Other algorithms perform well, and the division results are reasonable. On the ARI metric, the results of the algorithms such as CDID, FluidC, and LPA are very low and almost 0, and the FG and Louvain algorithms have relatively high *ARI* values. The performance of the IACD algorithm in large-scale real networks is satisfactory. Although the LPA algorithm has a best detection result, it is an unstable algorithm. The FluidC algorithm needs the number of real communities, but for most networks in the real world, we don’t know so much real information. The advantage of the IACD algorithm is that it does not require parameter settings and the segmentation results are stable. The quality of the segmentation is high and satisfactory.

#### 4.4.2. Performance Evaluation on Synthetic Networks

**Figure 4:**
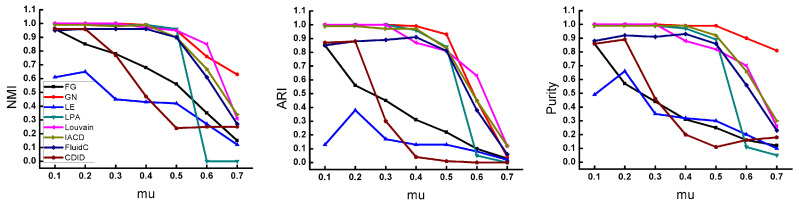
Results of different algorithms on synthetic networks with varying mix parameters.

To further compare the performance of different algorithms, we generate the LFR network by changing the mixing parameter mu, and the result shown in Figure 4. When the mixing parameter mu changes from 0.1 to 0.5, except for the CDID, LE, and FG algorithms, the quality of the community detected by other algorithms is very high on each metric. Among them, the IACD, GN, Louvain, and LPA algorithms remain high, and the FluidC also perform well. However, with the value of mu increases, the community structure becomes more and more difficult to distinguish, and the quality of the communities detected by these algorithms starts to decline. Specifically, when mu is 0.6, the LPA algorithm drops sharply, and each metric is almost 0, the GN algorithm performs well on the *NMI* and *Purity* metrics. The gap between the IACD algorithm and the Louvain algorithm is small when mu is 0.6, and the *NMI* and *Purity* of the IACD are slightly higher than the Louvain algorithm when mu is 0.7. Louvain algorithm is a stable community discovery algorithm commonly used in industry. Compared with Louvain, the IACD algorithm proposed in this paper has good performance when the mixing parameter mu changes on each metric.

**Figure 5:**
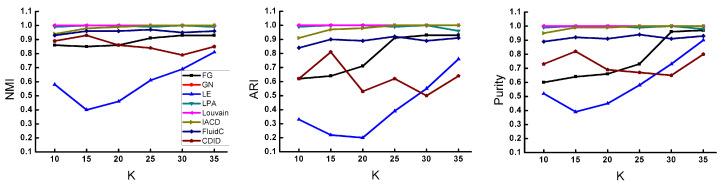
Results of different algorithms on synthetic networks with varying average degree.

Figure 5 shows the performance of these comparison algorithms on the LFR benchmark network when the average degree <k> changes from 10 to 35. When the average degree <k> is smaller, the community structure becomes more difficult to distinguish. It can be seen from the figure that the three algorithms of FG, CDID, and LE have a large fluctuation on the three metrics, when *K* is small, and the quality of dividing communities is not high. Among them, the FluidC and FG algorithms gradually increase the value of the index with the increase of *K*, but they still cannot reach the highest value when the structure of the community is very clear. The IACD algorithm we proposed is greater than 0.9 on each metric when *K* is small and has little difference from GN and Louvain algorithms. As the *K* increases, it quickly reaches the highest value with almost no change on each metric. Although the value of each metric has been stable at the maximum value at the beginning of the LPA algorithm, when *K* is 35, it obviously decreases. In general, the IACD algorithm has a good performance when *K* is small, and the community structure is not obvious. With the continuous clear structure of the community, the division result is stable and of high quality.

**Figure 6:**
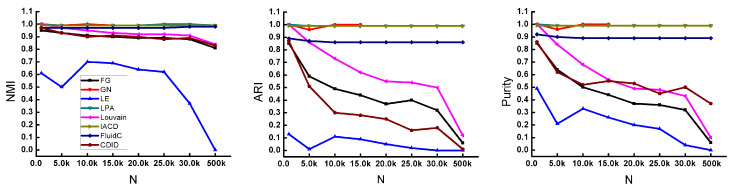
Results of different algorithms on synthetic networks with varying numbers of nodes.

Figure 6 shows the performance of these comparison algorithms on the LFR benchmark network when the number of nodes *N* changes from 1000 to 30,000. Due to the high time complexity of the GN algorithm, when the number of nodes *N* is 20,000, the results cannot be obtained for three days, the latter part is not shown in the figure. Except for the LE algorithm on the *NMI* metric, the other algorithms have achieved high values and are relatively stable. The three algorithms, LPA, IACD, and FluidC, are slightly higher than the Louvain, CDID, and FG algorithms. On the *ARI* and *Purity* metrics, the differences in these comparison algorithms can be clearly seen. Among them, Louvain, FG, and CDID algorithms show a significant downward trend, and the CDID algorithm fluctuate greatly. The value of the two metrics of the FluidC algorithm is basically stable at 0.9. The IACD algorithm and the LPA algorithm perform extremely well, and they have remain at the highest value and are very stable.The last point on the abscissa represents a large-scale dataset with a node size of 500,000. On *NMI*, Louvain, FG, and CDID algorithms decreased slightly, while LE decreased significantly, and *NMI* is almost 0. It can be clearly seen on *ARI* and *Purity* that FG, Louvian, LE, and CDID algorithms have low community detection quality on this dataset and a significant downward trend. While, the three algorithms, LPA, FluidC, and IACD, have a high detection result, and the trend remains stable. Among them, LPA and IACD have reached the highest value.

In synthetic networks, when the number of nodes is small, the quality of the partitioning effect of GN algorithm performs well. Due to its high time complexity, it cannot run on large-scale networks.The LPA algorithm performs very well when the mixing parameter mu is small. When the mixing parameter is greater than 0.5, the detection quality drops sharply. It achieves excellent performance in two sets of experiments with changes in average degree and node size, but the results detected by the algorithm itself are unstable due to the principle of label propagation. The FluidC algorithm performes well in three sets of experiments, and the detection results are stable. However, the algorithm itself needs the number of real communities, which has a lot of limitations. Louvain algorithm is a community detection algorithm commonly used in industry. The detection results are stable and effective in multiple experiments. When the mixing parameter mu and the node number are very large, the quality of the community detected by the IACD is higher than the Louvain algorithm. The CDID, LE, and FG algorithms have relatively large fluctuations in the detection results of the community in the three sets of experiments, and when the community structure is obvious, the detection results are relatively unsatisfactory.

## 5. Conclusions and Future Work

In this article, we draw on the law of universal gravitation in physics, combine it with the influence of nodes in complex networks, and propose a new community detection algorithm. First, the IACD algorithm re-expresses the importance of all nodes in the network, considering the node’s own degree value and the network location to make it more reasonable, and performs normalization processing. Then, based on the gravitational interaction relationship between celestial bodies in physics, we measure the gravitational relationship between the nodes, and each node forms an attractive node pair with the node that has the most attractive to it, and finally completes the community structure division. The IACD algorithm is concise and reasonable, and easy to understand. It performs well on real-world networks and different kinds of synthetic networks and the partition results are better than many classic algorithms and recently proposed algorithms. Besides, the algorithm execution process does not require parameter setting, which has low time complexity and stable result.

Actually, there is much room for improvement based on this algorithm. In the future, we will set out to make improvements in two directions. First, the algorithm proposed in this paper is oriented towards undirected unweighted networks, which is a simpler network structure. There are many networks in the real world with weighting relationships and directionality among nodes, which is more complex compared to undirected unweighted networks. How to improve the existing algorithms so that they can be applied to weighted and directed networks is also the next research direction. Secondly, the node influence calculation method used in this article combines degree centrality and the position of the node in the network, since there have been many interesting and effective node influence calculation methods proposed in recent years, how to choose the appropriate node influence calculation method is also a worthy direction to explore. 

## Figures and Tables

**Table 1:** Summary of the definitions.

Symbol	Definition
*n*	The number of nodes (*n* = |V|)
*e*	The number of edges (*e* = |E|)
Deg(v)	The degree of node *v*
N(v)	The neighborhood of node *v*
N2(v)	The second-order neighborhood of node *v*
KScore(v)	The k-core value of node *v*
IF(v)	The influence of node *v*
GIF(v)	The global influence of node *v*
Triangle(v,m)	The number of triangles formed by node *v* and *m*
P(v,m)	Coefficient of attraction of node *m* to node *v*
SL(v,m)	Shortest path length for node *v* and node *m*
Attr(v,m)	The attraction of node *m* to node *v*
Max_Strong(v)	The node with the greatest attraction to node *v*

**Table 2:** Node influence of simple networks.

Node	1	2	3	4	5	6	7	8	9	10	11	12	13	14	15	16
Deg (i)	5	4	4	5	3	4	4	2	5	4	5	4	4	2	2	3
KScore (i)	3	3	3	3	3	3	3	2	3	3	3	3	2	2	2	2
IF (i)	15	12	12	15	9	12	12	4	15	12	15	12	8	4	4	6
GIF (i)	1.0	0.8	0.8	1.0	0.6	0.8	0.8	0.27	1.0	0.8	1.0	0.8	0.53	0.27	0.27	0.4

**Table 3:** Node $v_{1}^{\prime }$s second-order neighbor attraction and shortest path length.

Node	2	3	4	5	6	9	10	11	12
Attr (i,j)	0.64	0.64	0.8	0.36	0.08	0.1	0.08	0.4	0.06
SL (i,j)	1	1	1	1	2	2	2	1	2

**Table 4:** Attractive node pairs of simple networks.

Node	1	2	3	4	5	6	7	8	9	10	11	12	13	14	15	16
MAX_Strong (i)	4	1	1	1	1	9	9	9	6	9	10	11	16	13	13	13

**Table 5:** The main parameters of LFR model.

Symbol	Definition
*N*	The number of nodes
<k>	Average degree
maxK	Maximum degree
minC	Minimum community size
maxC	Maximum community size
mu	Mixing parameter

**Table 6:** The statistics of six real-world complex networks: Node number |V|, edge number |E|, clustering coefficient <CC>, average degree <k> and number of communities #C.

Datasets	|V|	|E|	<CC>	<k>	#C
Karate	34	78	0.571	4.588	2
Dolphins	62	159	0.303	5.129	2
PolBook	105	441	0.48	8.4	3
AGBlogs	1222	33,428	0.36	27.335	2
UmbcBlog	404	2382	0.367	11.792	2
Amazon	334,386	925,872	0.397	5.53	75,149

**Table 7:** The relevant infomations of algorithms used for comparison.

Algorithm	Basic Principle	Time Complexity
LPA	Label propagation	O(n)
FG	Modularity optimization	O(e(n+e))
Louvain	Modularity optimization	$O(n\log^{2}n)$
GN	Betweenness	$O(e^{2}n)$
FluidC	Fluids interacting in an environment	O(e)
LE	Eigenvectors of matrices	$O(n^{2})$
CDID	Information dynamic	O(Lnlg<k>)
IACD	Internode Attraction	$O\left(n<k{>}^{2}\right)+O\left(e\right)$

**Table 8:** The results of real-world networks.

		Karate			Dolphins			PolBook			AGBlogs			UmbcBlog			Amazon		
		NMI	ARI	Purity	NMI	ARI	Purity	NMI	ARI	Purity	NMI	ARI	Purity	NMI	ARI	Purity	NMI	ARI	Purity
	FG	0.71	0.68	0.97	0.55	0.35	0.97	0.53	0.64	0.84	0.66	0.79	0.95	0.57	0.67	0.93	0.42	0.14	0.61
	GN	0.62	0.47	0.97	0.6	0.4	0.98	0.56	0.68	0.86	0.47	0.56	-	0.46	0.47	0.97	-	-	-
	LE	0.72	0.51	1	0.5	0.28	0.95	0.53	0.55	0.85	0.69	0.78	0.94	0.61	0.71	0.94	0.01	0.01	0.97
	LPA	0.69	0.68	0.90	0.65	0.49	0.97	0.55	0.65	0.85	0.66	0.74	0.91	0.48	0.54	0.90	0.52	0	0.99
	FluidC	0.71	0.74	0.95	0.56	0.46	0.98	0.54	0.6	0.88	0.7	0.79	0.93	0.61	0.71	0.90	0.51	0	0.98
	Louvain	0.61	0.46	0.97	0.56	0.34	0.97	0.51	0.55	0.85	0.64	0.77	0.95	0.55	0.55	0.95	0.47	0.13	0.98
	CDID	0.93	0.94	1	0.1	0	0.95	0.58	0.64	0.84	0.1	0	0.92	0.13	0	0.93	0.41	0	0.99
	IACD	1	1	1	0.63	0.44	0.97	0.61	0.67	0.85	0.7	0.8	0.95	0.62	0.71	0.95	0.51	0.01	0.98
